# SPAR – a randomised, placebo-controlled phase II trial of simvastatin in addition to standard chemotherapy and radiation in preoperative treatment for rectal cancer: an AGITG clinical trial

**DOI:** 10.1186/s12885-019-6405-7

**Published:** 2019-12-17

**Authors:** Michael B. Jameson, Kirsten Gormly, David Espinoza, Wendy Hague, Gholamreza Asghari, Grahame Mark Jeffery, Timothy Jay Price, Christos Stelios Karapetis, Michael Arendse, James Armstrong, John Childs, Frank A. Frizelle, Sam Ngan, Andrew Stevenson, Martinus Oostendorp, Stephen P. Ackland

**Affiliations:** 1 Waikato Hospital and Waikato Clinical Campus, University of Auckland, Hamilton, New Zealand; 2 Dr Jones & Partners, Eastwood, SA Australia; 3 NHMRC Clinical Trials Centre, University of Sydney, Sydney, Australia; 4 Bankstown-Lidcombe Hospital, Bankstown, Australia; 5 Christchurch Hospital, Christchurch, New Zealand; 6 Queen Elizabeth Hospital, University of Adelaide, Adelaide, Australia; 7 Flinders Medical Centre, Flinders University, Adelaide, Australia; 8 Waikato Hospital, Hamilton, New Zealand; 9 Consumer Advisory Panel, Australasian Gastro-Intestinal Trials Group, Sydney, Australia; 10 Regional Cancer and Blood Centre, Auckland District Health Board, Auckland, New Zealand; 11 Canterbury District Health Board, Christchurch, New Zealand; 12 Peter MacCallum Cancer Centre, Melbourne, Australia; 13 Royal Brisbane Hospital, Brisbane, Australia; 14 University of Newcastle, Lake Macquarie Private Hospital and Calvary Mater Newcastle Hospital, Newcastle, Australia

**Keywords:** Rectal cancer, Chemoradiation, Statins, HMG-coA reductase inhibitor, Tumour regression grading

## Abstract

**Background:**

Retrospective studies show improved outcomes in colorectal cancer patients if taking statins, including overall survival, pathological response of rectal cancer to preoperative chemoradiotherapy (pCRT), and reduced acute and late toxicities of pelvic radiation. Major tumour regression following pCRT has strong prognostic significance and can be assessed in vivo using MRI-based tumour regression grading (mrTRG) or after surgery using pathological TRG (pathTRG).

**Methods:**

A double-blind phase 2 trial will randomise 222 patients planned to receive long-course fluoropyrimidine-based pCRT for rectal adenocarcinoma at 18+ sites in New Zealand and Australia. Patients will receive simvastatin 40 mg or placebo daily for 90 days starting 1 week prior to standard pCRT. Pelvic MRI 6 weeks after pCRT will assess mrTRG grading prior to surgery. The primary objective is rates of favourable (grades 1–2) mrTRG following pCRT with simvastatin compared to placebo, considering mrTRG in 4 ordered categories (1, 2, 3, 4–5). Secondary objectives include comparison of: rates of favourable pathTRG in resected tumours; incidence of toxicity; compliance with intended pCRT and trial medication; proportion of patients undergoing surgical resection; cancer outcomes and pathological scores for radiation colitis. Tertiary objectives include: association between mrTRG and pathTRG grouping; inter-observer agreement on mrTRG scoring and pathTRG scoring; studies of T-cell infiltrates in diagnostic biopsies and irradiated resected normal and malignant tissue; and the effect of simvastatin on markers of systemic inflammation (modified Glasgow prognostic score and the neutrophil-lymphocyte ratio). Trial recruitment commenced April 2018.

**Discussion:**

When completed this study will be able to observe meaningful differences in measurable tumour outcome parameters and/or toxicity from simvastatin. A positive result will require a larger RCT to confirm and validate the merit of statins in the preoperative management of rectal cancer. Such a finding could also lead to studies of statins in conjunction with chemoradiation in a range of other malignancies, as well as further exploration of possible mechanisms of action and interaction of statins with both radiation and chemotherapy. The translational substudies undertaken with this trial will provisionally explore some of these possible mechanisms, and the tissue and data can be made available for further investigations.

**Trial registration:**

ANZ Clinical Trials Register ACTRN12617001087347.

(www.anzctr.org.au, registered 26/7/2017)

**Protocol Version:** 1.1 (June 2017).

## Background

### Summary of clinical condition and current treatments

Colorectal cancer (CRC) is common: 3016 cases were diagnosed in New Zealand (NZ) in 2012 with 1283 deaths [1] and rectal cancer represents about one-third of all colorectal cancers in NZ [2]. In Australia, 5114 rectal cancer cases were diagnosed in 2011 with 2018 deaths in 2012 [3]. Rectal cancer usually presents with locally-advanced T3 disease that requires ‘short course’ radiotherapy (SCRT) or, more commonly, ‘long course’ preoperative chemoradiation (pCRT – in which either infusional 5-fluorouracil (5FU) or oral capecitabine are administered concurrently with radiotherapy) for 5–6 weeks before surgery, and often adjuvant post-operative chemotherapy. While these advances in the management of resectable rectal cancer have reduced local relapse to < 10% in most patients, those with higher tumour stage, or evidence on staging MRI scan of invasion of local nodes, mesorectal fascia or blood vessels, have substantially higher local relapse rates and poorer overall survival (OS) [4]. In addition, distant relapse still occurs in 25–30% of patients, with most dying within 5 years [5]. Adding more drugs (such as oxaliplatin or irinotecan) to pCRT increases toxicities but with no improvement in cancer outcomes [6]. Other strategies are being explored in phase 2 and 3 trials but none have yet changed the standard of pCRT (or, less commonly, SCRT).

Unfortunately, the majority of patients (about 60%) with high-risk tumours have poor responses of their tumour to pCRT, and this group have double the risk of relapse compared to good responders [7]. Furthermore about 10% of surviving patients suffer from long-term significant bowel toxicity from RT. [5, 8] There is a clear need for improved efficacy and reduced toxicity in the large number of rectal cancer patients treated with pCRT every year in NZ and Australia.

### Summary of findings from pertinent pre-clinical studies and clinical trials

Statins offer the opportunity to improve outcomes in the treatment of rectal cancer. A Danish population study of 295,925 cancer cases of all types revealed that the use of statins significantly improved overall survival (OS) and specifically in those with CRC (HR 0.79, 95% CI 0.74–0.85) [9]. Similarly, a registry study of 10,762 CRC patients from Taiwan reported that, on multivariate analysis, cancer-specific survival was independently and significantly improved in statin users (HR 0.72 *p* < 0.001) [10]. More recently a population-based cohort study of 7657 patients with CRC in the United Kingdom showed that statin use improved cancer-specific survival (HR, 0.71; 95% CI, 0.61–0.84) and all-cause mortality (HR, 0.75; 95% CI, 0.66–0.84) [11].

Preclinical studies have elaborated effects on cell signalling pathways that may contribute to better cancer outcomes with statins, many of which are independent of cholesterol metabolism [12]:

- generation of pro-apoptotic, growth-inhibitory and pro-differentiation responses in tumours;
- inhibition of angiogenesis, invasion and metastasis;
- reducing inflammation and inhibiting radiation (RT)-induced gut and skin toxicities while radiosensitising tumour cells and maintaining tumour control compared to RT alone [13–20].

This correlates with retrospective clinical studies in which patients taking statins during RT or chemo-RT for rectal, bladder or prostate cancer had significantly higher rates of pathological complete response (CR), local control and progression-free survival, respectively [21–25]. The findings in three published retrospective studies using pCRT in rectal cancer patients were:

- in 407 patients at the Cleveland Clinic, favourable pathological regression was seen in 65.7% of statin users vs. 48.7% of others, *p* = 0.004 (multivariate OR 2.25; 95% CI 1.33–3.82) [22].
- multivariate analysis of 891 Canadian patients from multiple centres showed a significantly higher pathological CR rate in statin users (OR 1.7, 95% CI 1.04–2.89, *p* = 0.044) [23];
- in 349 patients from Memorial Sloan-Kettering Cancer Center pathological CR was higher in statin users (30% vs. 17%), with multivariate OR 4.2 (95% CI 1.7–12.1; *p* = 0.003) [24].

Furthermore, in a prospective observational study of 308 patients treated with radical pelvic RT at the Royal Marsden Hospital, London, those taking statins had significantly reduced RT-induced bowel toxicity, both during treatment (*p* = 0.04) and 1 year later [26].

In contrast two smaller studies have not shown an apparent benefit. A retrospective New Zealand study of 129 rectal cancer patients showed neither reduced acute toxicity nor improved pathological CR rates in the 23% of patients who took statins during pCRT [27]. A US single-arm phase 2 trial recruited 53 prostate cancer patients to take lovastatin 20–80 mg daily for 1 year starting during external beam RT and/or brachytherapy to prevent late RT-induced rectal injury [28]. Persistent gastrointestinal symptoms at 2 years were seen in 32% of patients (grade 2 in 6%), which did not meet the primary endpoint. Furthermore no benefit from statins was seen in a meta-analysis of surgery for prostate cancer [25, 29], nor in a trial of adjuvant chemotherapy for colon cancer [30].

### Summary of the known and potential risks and benefits to human participants

The statin chosen for this trial, simvastatin (SIM), is a well-known and widely available HMG-CoA reductase inhibitor commonly used in the treatment of hypercholesterolaemia and ischaemic heart disease. In retrospective studies it reduces recurrence in breast cancer patients [31] and preclinical studies have demonstrated its beneficial interactions with RT. [32, 33] It is very well-tolerated in the majority of patients with < 2% of patients in clinical trials discontinuing simvastatin due to adverse events. The most common side effects include abdominal pain, diarrhoea, indigestion, and weakness. Rarer side effects include joint pain, memory loss, myalgia, and muscle cramps. In patients taking statins long-term there have been reports of hepatitis, rhabdomyolysis and myositis however these complications are rare (< 1% of patients). Serious allergic reactions to simvastatin are also rare. 40 mg daily is the highest dose well-tolerated [34].

### Rationale for trial endpoints

#### mrTRG and pathTRG

MRI-based tumour regression grading (mrTRG), a 5-point system validated by the MERCURY group [35], is used for the primary endpoint in this trial as it permits much more sensitive and reliable preoperative assessment of tumour regression following pCRT in rectal cancer patients than other methods used currently (endoscopy, CT, PET-CT, endoluminal ultrasound or routine MRI) [36, 37]. mrTRG has proven to be a good predictor of pathological tumour regression grading (pathTRG) after pCRT [38], as well as independently predicting DFS and OS [39]. In a separate study, mrTRG identified ten times as many pathological CR patients as clinical inspection of the tumour following pCRT, with no compromise of the false-positive rate [37].

The rate of favourable (grades 1–2) mrTRG is the primary endpoint for the SPAR trial, based on three recently-published pCRT rectal cancer trials [40, 41]. In the MERCURY-II trial [40] favourable (grades 1–2), intermediate (grade 3) and unfavourable mrTRG (grades 4–5) had 3-year DFS of 82, 72 and 61% respectively (G Brown, personal communication). The phase 2 EXPERT and EXPERT-C trials evaluated neoadjuvant chemotherapy followed by pCRT [41]. On pooled analysis of these trials, mrTRG performed 4 weeks after completion of pCRT was evaluable in 85.5% of 269 patients; favourable (mrTRG 1–2), intermediate (mrTRG 3) and unfavourable (mrTRG 4–5) outcomes were seen in 41.7, 30.9 and 27.4%, respectively. pathTRG was evaluable in 86.9% of 244 resected patients with favourable, intermediate and unfavourable scores seen in 35.4, 29.7 and 34.9% respectively. Favourable mrTRG was independently associated with PFS (HR 0.37, *p* < 0.001) and OS (HR 0.44, *p* = 0.006) [41].

While the mrTRG training provided in SPAR has been shown to achieve moderate-to-excellent agreement between expert and training radiologists [42], it is important to show independently in the SPAR trial that mrTRG can be successfully and reproducibly performed in multiple centres, and shows a strong correlation with pathTRG and clinically-important cancer outcomes.

While pathological CR with pCRT is associated with the best clinical outcomes, and is commonly considered the “gold standard”, the spectrum of response on standardised pathological tumour regression grading (pathTRG) systems correlates with DFS and overall survival (OS), and informs prognosis in the full spectrum of patients, not just the small minority with pathological CR. Thus a 4-tier system has been widely adopted, including in Australasia [43], but a 3-tier system derived from this (grouping the two most favourable grades) shows greater reproducibility (interobserver agreement κ = 0.84) and is recommended [44].

SPAR will provide independent validation of the correlation of mrTRG with pathTRG and cancer outcomes, as well as evaluating the reproducibility of mrTRG assessment by NZ and Australian radiologists (not yet commonly used) and pathTRG by pathologists (standard practice).

Early surrogates for tumour response to pCRT are being investigated in clinical trials, particularly for their potential to modify the extent of surgery, or possibly avoidance of surgery in those who achieve a radiological CR [36]. The importance of this is three-fold: firstly, mrTRG can assist surgeons in planning the extent of surgery based on the response to pCRT; for example, this could change whether a patient needs a permanent stoma or reduce the risk of positive circumferential resection margins. Secondly, some patients who appear to have clinical CR after pCRT are electing to avoid surgery, based on much less precise methods of assessing residual disease than mrTRG, so using mrTRG can improve the precision of assessment of likely pathological CR [36, 37]. Thirdly, the poor outcomes of patients with an unfavourable response to pCRT has led to proposals to use mrTRG to identify this group in clinical trials and evaluate if adding other treatment (such as different chemotherapy or biological therapy) prior to surgery can improve cancer outcomes (e.g. The TRIGGER Study; clinicaltrials.gov No. NCT02704520).

#### Timing of post-pCRT MRI

In the SPAR study, the second MRI is scheduled at 6–8 weeks after pCRT to allow for surgery at 7–12 weeks. Surgery is now commonly delayed to 10–12 weeks after completion of pCRT in anticipation of improved tumour regression with additional time after pCRT [45]. There are conflicting reports as to whether this is the case, based on pathological CR rates [46–48]. However, while the pathological changes in the tumour following pCRT are expected to evolve over many weeks, this may not improve DFS or PFS as these outcomes are most likely determined by the inherent tumour sensitivity to pCRT. This is supported by a retrospective Korean study of 1786 patients treated with pCRT for locally-advanced rectal cancer, in whom pathological CR rates were highest when surgery was performed 5–10 weeks after pCRT; those who had resection delayed to > 7 weeks after pCRT had significantly higher pathological CR rates but no difference in relapse-free or overall survival [49]. Of concern, a French prospective randomised controlled trial of surgery at 7 or 11 weeks after pCRT in 265 rectal cancer patients showed no significant difference in the primary endpoint of pathological CR rates but there was significantly higher post-operative morbidity and poorer quality of mesorectal excision in the 11 week group, possibly due to greater RT-induced fibrosis [47]. A retrospective US study in 6397 patients evaluating time between RT and resection for rectal cancer found that an interval > 60 days was significantly associated with inferior survival, lower rates of sphincter-preserving surgery and an increased rate of positive surgical margins [48]. This suggests that surgery could be optimally performed earlier than commonly practised currently, and SPAR will accommodate this range.

#### Translational endpoints

This trial is an important opportunity to identify whether assessment of systemic inflammation (reflected in the modified Glasgow prognostic score, mGPS) and the local inflammatory response (through characterising infiltrating lymphocytes) could be an important translational research component of a subsequent phase III trial of statins in rectal cancer patients. Additionally, it will allow us to evaluate the impact of SIM on the relationship between specific T-cell infiltrates in pre-pCRT biopsies and pathTRG in the resected tumours, and with normal tissue inflammation post-pCRT.

It is long-recognised that a local inflammatory response with infiltration of T-lymphocytes into CRC carries a better prognosis, independent of tumour stage [50, 51]. Conversely preoperative systemic inflammation, reflected in the blood neutrophil/lymphocyte ratio (NLR) or serum c-reactive protein (CRP) and albumin levels (summarised in the modified Glasgow Prognostic Score), correlates with worse prognosis, independent of stage and preoperative therapy [52]. This adverse prognostic relationship persists with elevated mGPS 3–6 months postoperatively [52]. While statins are anti-inflammatory [12] and reduce the NLR in patients with high cholesterol [53], it is not known whether they lower the NLR or mGPS in cancer patients.

The Immunoscore is a recently-validated test that characterises T-cell subsets infiltrating into the centre of the tumour and at the invasive margin of CRC by immunohistochemistry (IHC) for CD3 and CD8; higher scores correlate with a lower risk of relapse and improved DFS and OS, independently of stage [54, 55]. While the Immunoscore was validated in rectal cancer patients who had primary surgery, it cannot be applied to those patients who received pCRT because identification of central and margin regions is compromised due to tumour regression and fibrosis [56]. However, in diagnostic rectal biopsies taken prior to pCRT prominent infiltration of CD3+ and CD8+ T-cells strongly correlated with pathological CR rates following pCRT (56).

Statins have complex effects on T-cell biology, including induction of regulatory T-cells (Tregs), their migration into tumours and inhibition of the induction of Th1 and Th17 cells [57]. Tregs, which dampen immune responses, can differentiate into effector Tregs (eTregs); these display markers of both immune suppression and activation [58] and are associated with a positive patient outcome in CRC [59]. However, Treg populations in lymph nodes do not correlate with patient outcome, unlike their presence in the primary colorectal tumour [60].

### Overall purpose

The overall purpose of this trial is to determine the effect of SIM on outcomes of pCRT for rectal cancer. Outcomes will be evaluated both by tumour regression as well as the tolerability of pCRT, estimating the size of the benefit by MRI-based assessment as well as pathological assessment of tumour regression. In addition, we will examine the biological mechanisms involved. The study will also assess the reproducibility of assessing mrTRG and pathTRG by radiologists and pathologists in Australia and New Zealand. Furthermore, the information provided by the post-CRT mrTRG could influence the intended surgical plan and optimise timing of surgery depending on tumour response to pCRT.

A positive outcome, either improved tumour regression or reduced toxicity from pCRT or both, would lead to a larger phase III trial to confirm these findings. It may also lead to evaluation of statins in prospective trials in many other settings where radiation is used in cancer treatment. This trial is an important opportunity to identify whether the addition of SIM to pCRT significantly modulates systemic inflammation (reflected in the NLR and mGPS) and the local inflammatory response (through characterising infiltrating immune cells). If so, it would inform the inclusion of these translational research components in a subsequent phase III trial of statins in rectal cancer patients. It will also allow us to evaluate the impact of SIM on the correlation of T-cell infiltrates in pre-pCRT biopsies with pathTRG in the resected tumours, and with normal tissue inflammation after pCRT.

## Methods/design

SPAR is a randomised phase 2 study with the overall aim to evaluate the effect of SIM on efficacy and toxicity of pCRT in rectal cancer patients, and on systemic and local inflammatory responses. Recruitment of 222 patients is required to address the primary objective. The study will recruit patients from participating AGITG hospitals/institutions in Australia and New Zealand.

Primary Objective:

The primary objective is to compare rates of favourable (grades 1–2) mrTRG (by central review) following pCRT with SIM versus with placebo, considering mrTRG in 4 ordered categories: 1, 2, 3, 4–5 (proportion of patients with favourable mrTRG in SIM and placebo groups). mrTRG will be assessed by comparison of the MR scan taken after pCRT with the scan before pCRT, which will be performed using the MERCURY protocol (38), and images analysed as described [38].

Secondary Objectives:

Secondary objectives are to compare between the SIM and placebo groups treated with pCRT: 1) The rate of favourable (grades 1–2) pathTRG in resected tumours by central review (proportion of patients with favourable pathTRG at surgical resection); 2) The incidence of > grade 2 acute GI and non-GI adverse events, assessed using CTCAE version 4.03; 3) The incidence of late GI adverse events; 4) Compliance with intended pCRT (proportion of patients completing > 90% of planned pCRT without dose reductions or delays); 5) Compliance with trial medication (proportion of patients receiving > 90% of the planned trial medication); 6) The proportion of patients undergoing surgical resection post-pCRT; 7) 3-year local recurrence (LR) rate, disease-free survival (DFS) and cancer-specific survival (CSS) (3-year LR rate, DFS and CSS); 8) the pathological scores determined by the central pathologist for radiation colitis in irradiated rectum in the resected specimen (radiation colitis scores).

Translational Science Objectives:

A number of correlative/translational objectives are intended. We aim to determine in the total trial population: 1) The association between mrTRG and pathTRG grouping (association between mrTRG and pathTRG grouping); 2) The inter-observer agreement between site radiologists and a central radiologist on mrTRG scoring (inter-observer agreement on mrTRG scoring); 3) The inter-observer agreement between site pathologists and a central pathologist on pathTRG scoring (inter-observer agreement on pathTRG scoring). In addition we aim to compare between the SIM and placebo groups treated with pCRT: 1) The association between CD3+ and/or CD8+ T-cell infiltrates in the tumour in the pre-pCRT diagnostic biopsies and pathTRG (association between T cell infiltrates and pathTRG); 2) the intensity and distribution of subsets of infiltrating T-cells in irradiated normal and malignant tissue in the resected specimen; 3) The influence of SIM on systemic inflammation, assessed with the mGPS and the NLR (mGPS and NLR).

### Design

**Fig. 1:**
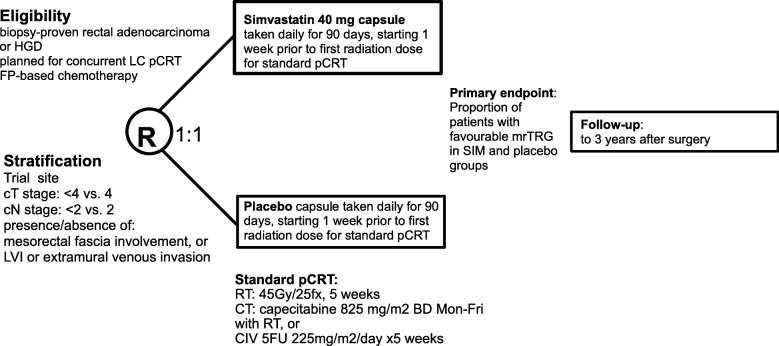
SPAR SPIRIT diagram. Abbreviations: HGD; high grade dysplasia. LC; long course. pCRT; preoperative chemoradiation. FP; fluoropyrimidine. cT/cN; clinical stage. LVI; lymphovascular invasion. ®; randomisation. Gy; gray. fx; fraction. BD; twice daily. 5FU; 5-fluorouracil. mrTRG: magnetic resonance assessed tumour regression grade

SPAR is a randomised, double-blind, placebo-controlled, multicentre phase II trial. Eligible patients will be allocated to one of two treatment groups (SIM or placebo) in a 1:1 ratio (Fig. 1: SPIRIT diagram).

Treatment allocation will be balanced using minimisation for the following characteristics:

- trial site
- AJCC clinical T stage (< 4 vs. 4)
- AJCC clinical N stage (< 2 vs. 2)
- the presence of either mesorectal fascia involvement (tumour margin within 1 mm of the fascia) or lymphovascular space invasion (including extramural venous invasion) on MRI

### Eligibility criteria

The target population is adult patients with biopsy-proven rectal adenocarcinoma (or high-grade dysplasia on biopsy with radiological evidence of invasive tumour) planned for concurrent long-course pCRT using a standard fluoropyrimidine-based chemotherapy schedule.

#### Inclusion criteria

1. Males or females with biopsy proven rectal adenocarcinoma, or high-grade dysplasia with radiological evidence of invasive tumour.
2. Distal border of the tumour is below the peritoneal reflection as assessed by MRI scan.
3. Age ≥ 18 years.
4. Clinical TNM tumour staging is T2–4 N0–2 M0 after staging investigations including CT scan of chest, abdomen and pelvis and pelvic MRI scan. Patients with resectable M1 (e.g. oligometastatic liver or lung) disease who are being treated with curative intent can be eligible, following approval from the SPAR Study Chair.
5. Planned for concurrent long-course pCRT using fluoropyrimidine-based chemotherapy
6. Radiologically measurable disease on baseline pelvic MRI scan.
7. Adequate bone marrow, liver and renal function (platelets > 100 × 10^9^/L, neutrophils > 1.5 × 10^9^/L, ALT/AST < 3 x ULN, bilirubin < 1.5 x ULN, estimated creatinine clearance > 50 ml/min).
8. Trial treatment planned to start within 28 days of randomisation.
9. Diagnostic biopsy of rectal tumour is available for histological substudies.
10. Willing and able to comply with all trial requirements.
11. Signed, written informed consent for the main trial.

#### Exclusion criteria

1. Contraindications or hypersensitivity to statins, fluoropyrimidine chemotherapy or radiotherapy
2. Patients planned to receive oxaliplatin or biological agents (e.g. cetuximab) as part of pCRT
3. Taking statins in the 6 weeks before planned start of pCRT
4. Predicted life expectancy of less than 3 years
5. Prior pelvic or rectal radiotherapy
6. History of another malignancy within 5 years prior to registration (not including adequately treated carcinoma-in-situ, basal cell carcinoma of the skin, squamous cell carcinoma of the skin, or superficial transitional cell carcinoma of the bladder). Patients with a history of other malignancies are eligible if they have been continuously disease free for at least 5 years after definitive primary treatment
7. Concurrent illness, including severe infection that may jeopardise the ability of the patient to undergo the procedures outlined in this protocol with reasonable safety
8. Serious medical or psychiatric conditions that might limit the ability of the patient to comply with the protocol
9. Pregnancy, lactation, or inadequate contraception.

### Treatment

Simvastatin or placebo is the trial intervention. Concurrent preoperative chemoradiation (pCRT) using fluoropyrimidine-based chemotherapy and radiotherapy is required standard concomitant treatment (Fig. 1).

One simvastatin 40 mg capsule or one matching placebo capsule taken orally each evening, will commence 7 (+/− 3) days prior to pCRT and continued for 90 consecutive days. Subject compliance with the trial treatment will be determined at protocol-specified assessments by questioning the participant and a formal count of the capsules returned at completion of study treatment (6 weeks after pCRT).

Trial treatment (SIM or placebo) will be permanently discontinued if progressive disease (PD) is documented, unacceptable treatment-related toxicity occurs, a delay of pCRT of > 28 days occurs, the clinician believes that continuation of trial treatment is not in the patient’s best interest, or the patients fails to comply or declines further treatment.

### Radiation therapy

It is recommended to follow eviQ Rectal (Neoadjuvant EBRT Chemoradiation Pre-operative Long Course) Guidelines version 2 or later (www.eviq.org.au).

Clinical Target Volume A (CTV A) is defined as per the recent international consensus guidelines [61]. The Planning Target Volume (PTV) A margin should be 0.7 to 1.0 cm, except at skin, where planning system requirements mandate it be trimmed to 2–5 mm within the skin surface. The dose to PTV A is 45Gy in 25 fractions, 1.8Gy per day, 5 days per week.

Clinical Target Volume B (CTV B) includes the mesorectum and pre-sacral region at involved levels with a 1–2 cm margin cephalad and 1 to 2 cm on gross tumour within the rectum. The PTV B margin should be 0.7 to 1.0 cm, except at skin, where planning system requirements mandate it be trimmed to 2–5 mm within the skin surface.

For 3-dimensional conformal radiation therapy (3DCRT), a tumour boost of 5.4Gy at 1.8Gy per fraction to PTV B (50.4Gy cumulative including contribution from PTV A) is required for patients with T3 tumours. A boost dose up to 10.8Gy at 1.8Gy per fraction (55.8Gy cumulative, including contribution from PTV A) is allowed for patients with T4 fixed cancer and high-risk T3 tumours.

For intensity modulated radiation therapy (IMRT) or volumetric modulated arc therapy (VMAT), simultaneous boost technique is recommended. Total cumulative dose to PTV B is to be 50 Gy.

Permission for other radiation techniques aiming to deliver equivalent radiation dose should be obtained in advance from Trial Management Committee.

### Chemotherapy

The accepted chemotherapy and dosing regimens [6] are one of:

- capecitabine 825 mg/m^2^/day PO BID 5 days a week on days of RT administration
- capecitabine 825 mg/m^2^/day PO BID 7 days a week for the duration of RT
- 5-fluorouracil 225 mg/m^2^/day via continuous venous infusion for the duration of RT
- Other therapies and/or dosing regimen that have been accepted as standard of care in Australia and New Zealand may be allowed following agreement from the Study Chair

A maximum BSA of 2.2 m^2^ is recommended for dosing of fluoropyrimidine-based chemotherapy. Clinicians must pre-specify which schedule they will be using for each participant. Once allocated, the patients must then adhere to the specified schedule throughout the treatment period, unless modified for safety reasons.

### Dose modifications and supportive therapies

Patients should be managed with pCRT according to institutional protocols, including dose modifications and delays for treatment-related toxicities. Recommendations are provided for guidance on management of toxicities related to pCRT and are defined in the protocol. If pCRT is discontinued, trial treatment should continue unless unacceptable trial treatment toxicity is observed. Other concomitant medications and supportive therapies are permitted, and there is a list of prohibited medications (drugs that may interact with the trial drug) in the detailed protocol.

### Surgery

Patients will undergo resection of their rectal cancer at a time recommended by their surgeon (generally 7–10 weeks after completion of pCRT). Patients with an excellent clinical and radiological response may be observed under a “watchful waiting” programme if agreed with their surgeon.

Surgery may include open, laparoscopic, robotic or transanal total mesorectal excision approach. This will include both restorative low anterior resection, or abdomino-perineal resection with permanent end-colostomy.

### Post-operative management

Treatment after discontinuation of trial treatment is at the discretion of the patient’s clinician. Adjuvant chemotherapy may be administered on the recommendation of the treating clinician and its use will be recorded.

Blood tests will be performed on the first day of pCRT, prior to starting chemotherapy, to assess the impact of trial medication on the mGPS and NLR. Clinical assessments including IBDQ-B questionnaire will be conducted on Trial Weeks 3, 5 and 7 [62].

Week 13 assessments will be performed 6 weeks (+/− 7 days) after completion of pCRT and prior to surgery. An MRI scan for mrTRG must be completed 6–8 weeks after completion of pCRT and must be prior to surgery. The postoperative visit will include assessment of length of hospital stay after surgery and any readmission data. Subsequent follow-up is annually.

### Data to be collected

The study will collect data on: baseline patient and tumour status; treatment delivery; baseline and post-pCRT MRI assessments; clinical and laboratory toxicity assessments; baseline and resection histopathology; followup data for PFS and OS (Table 1).

**Table 1:** Schedule of Assessments

Treatment Period – Trial Week											
	Baseline / screening	1	2	3	4	5	6	7	13	4–6 weeks post-op	Follow up
Informed consent	X										
History	X									X	
Physical exam ^b^	X			X		X		X		X	
Trial medication start		X									
Preoperative CRT			X	X	X	X	X	X			
Concomitant meds	X			X		X		X	X		X
Trial med compliance				X		X		X	X		
Adverse events				X		X		X	X		X
Haematology	X		X	X	X	X	X	X	X		
Chemistry incl. CRP	X		X	X	X	X	X	X	X		
CEA	X										X
Serum pregnancy test	X										
Resected rectal tissue										X	
CT scan	X										X
Pelvic MRI	X								X ^o^		
Lower GI endoscopy	X										X

### Statistical methodology

#### Sample size

The primary endpoint of this trial is rates of favourable mrTRG (grades 1 or 2). This will be analysed using ordinal regression with 4 ordered categories of mrTRG (1, 2, 3 and 4–5) to provide greater sensitivity. Based on published data using MRI 6–8 weeks after pCRT, the expected rate of mrTRG in the control group is 9% grade 1, 39% grade 2, 20% grade 3 and 32% grades 4–5. A 35% relative increase in favourable mrTRG would be a worthwhile difference to inform a phase III trial.

Based on an ordinal proportional odds assumption, a sample size of 222 patients (111 treated with SIM and 111 controls) will have > 80% power to detect a change in mrTRG rates to 17% grade 1, 48% grade 2, 16% grade 3 and 19% grades 4–5 with 95% confidence.

#### Statistical analysis

Intention-to-treat analysis of results will be the primary analysis. In addition, an exploratory per protocol analysis will also be conducted which will exclude ineligible patients, those not taking trial medication when starting pCRT and those who withdraw their consent to participate prior to response evaluation.

The primary endpoint and all secondary endpoints expressed as proportions will be estimated, together with corresponding 95% confidence intervals based on exact binomial distributions. Kaplan-Meier curves will be calculated for all time-to-event endpoints. Rates at specific points in time (e.g. 3-year local recurrence rate) will be estimated from these Kaplan-Meier curves.

Inter-observer agreement is defined as the degree of agreement in mrTRG (or pathTRG) results when reviewed by the central radiologist (or pathologist) and the reporting site radiologists (or pathologists). Results for each of mrTRG and pathTRG will be reported separately in a 3X3 grid comparing favourable, intermediate, and unfavourable scores between central and site radiologists and pathologists respectively. Inter-observer agreement will be assessed using a weighted kappa statistic > 0.40 is defined as moderate agreement. This will be assessed for mrTRG after recruitment of 35 randomised patients to ensure procedural consistency and again at the conclusion of the trial.

Adverse events in each arm will be tabulated and graded according the NCI CTCAE version 4.03.

For analysis of translational objectives, association between CD3+ and CD8+ T-cell infiltrates in diagnostic rectal cancer biopsies and pathTRG following pCRT will be evaluated using the chi-square test for trend. The effect of SIM on subsets of T-cells in tumours and normal tissue will be evaluated by comparison of distribution of scores for each cell type in SIM and placebo groups using the chi-square test for trend. Changes in the NLR and mGPS over time will be analysed using repeated measures ANOVA.

#### Interim analyses and early stopping

No formal interim analysis for efficacy is planned but review of safety data by the AGITG Independent Safety and Data Monitoring Committee (ISDMC) is planned. No early stopping for larger-than-expected differences in mrTRG rates is planned because this endpoint is a putative surrogate for improved patient outcome. Demonstrating a significant difference in the clinically-important DFS and OS endpoints requires a much larger phase III trial.

### Trial governance and confidentiality

The study is conducted by the Australasian Gastrointestinal Trials Group, in conjunction with the NHMRC Clinical Trials Centre. Formal study oversight is by a Trial Management Committee and an Independent Data and Safety Monitoring Committee.

The study will be conducted according to the Note for Guidance on Good Clinical Practice (CPMP/ICH/135/95) annotated with TGA comments (Therapeutic Goods Administration DSEB July 2000) and in compliance with applicable laws and regulations. The study will be performed in accordance with the NHMRC Statement on Ethical Conduct in Human Research 2007, the NHMRC Australian Code for the Responsible Conduct of Research 2007, and the principles laid down by the World Medical Assembly in the Declaration of Helsinki 2008.

The study will be conducted in accordance with applicable Privacy Acts and Regulations. All data generated in this study will remain confidential. All information will be stored securely at the NHMRC Clinical Trials Centre, University of Sydney, and will only be available to people directly involved with the study and who have signed a Confidentiality Agreement.

## Discussion

Little progress has been made in the management of T3 rectal cancer in the last 10 years. The findings in several retrospective studies that statin use in patients undergoing pCRT appears to confer higher pathological regression rates, more pathological CR [22–24] and also lower RT toxicity [16, 22–24], led us to mount this phase II RCT. We chose to start statin therapy 1 week prior to pCRT to observe for clinical and biochemical effects independent of pCRT, and continue it for 3 months to allow maximum interaction with CRT. The use of a standard dose of simvastatin is pragmatic; 40 mg per day is the highest dose that is well tolerated. The evaluations are comprehensive, including comparative treatment-induced changes in MR scans, pathological evaluation of the resected specimen, and blood biochemical changes, as well as longer-term tumour outcomes (PFS and OS). With this sample size of 222 patients (111 simvastatin and 111 placebo), we should be able to observe meaningful differences in these parameters if simvastatin has any beneficial effect in the treatment of this disease.

If this study has a positive result, a larger RCT will be needed to confirm and validate the merit of statins in the preoperative management of rectal cancer, especially long-term clinical outcomes. Such a finding could also lead to studies of statins in conjunction with radiation and chemotherapy in a range of other malignancies, as well as further exploration of possible mechanisms of action and interaction of statins with both radiation and chemotherapy. The translational substudies undertaken with this trial will provisionally explore some of these possible mechanisms, and the tissue and data can be made available for further investigations.

This trial is an important opportunity to identify whether assessment of systemic inflammation (reflected in the neutrophil-lymphocyte ratio and the mGPS) and the local inflammatory response (through characterising infiltrating immune cells) could be important translational research components of a subsequent phase III trial of statins in rectal cancer patients. Additionally, it will allow us to evaluate the impact of SIM on the interaction of specific T-cell infiltrates in pre-pCRT biopsies with pathTRG in the resected tumours, and with normal tissue inflammation after pCRT.

Recent reports on the merits of mrTRG to assess effects of pCRT suggest that it is a valid endpoint to use in clinical trials [37–41]. This study will further validate mrTRG as an interim endpoint for assessing treatment benefits in rectal cancer, as well as allow exploration of newer MRI features that might refine the TRG assessment. All MR scans will be collected in digital format and can be made available to other groups for further research.

Surgery is now commonly delayed to 10–12 weeks after completion of pCRT in anticipation of improved tumour regression with additional time after pCRT. There are conflicting reports as to whether this is the case, based on pathological CR rates [46–48]. However, while the pathological changes in the tumour following pCRT are expected to evolve over many weeks, this may not improve DFS or PFS as these outcomes are most likely determined by the inherent tumour sensitivity to pCRT. This is supported by a retrospective Korean study of 1786 patients treated with pCRT for locally-advanced rectal cancer, in whom pathological CR rates were highest when surgery was performed 5–10 weeks after pCRT; those who had resection delayed to > 7 weeks later had significantly higher pathological CR rates but no difference in relapse-free or overall survival [49]. Of concern, a French prospective randomised controlled trial of surgery at 7 or 11 weeks after pCRT in 265 rectal cancer patients showed no significant difference in the primary endpoint of pathological CR but there was significantly higher post-operative morbidity and poorer quality of mesorectal excision in the 11-week group, possibly due to greater RT-induced fibrosis [47]. A retrospective US study (*n* = 6397) evaluating time between RT and resection for rectal cancer found that an interval > 60 days was significantly associated with inferior survival and rates of sphincter-preserving surgery and an increased rate of positive surgical margins [48]. This suggests that surgery could be optimally performed earlier than commonly practised currently. In this SPAR study, surgery is intended at 7–10 weeks after completion of pCRT. The data we collect may contribute to refinement of guidelines about surgery timing after pCRT.

## Data Availability

The study is currently accruing. Data and materials will be made available for further research upon request after the primary analysis has been presented and published.

## Abbreviations

5FU: 5-fluorouracil
AJCC: American Joint Committee on Cancer
CR: Complete remission/response
CRC: Colorectal cancer
CRP: C-reactive protein
CSS: Cancer-specific survival
CTV: Clinical target volume
DFS: Disease-free survival
EBRT: External beam RT
GI: Gastrointestinal
HMG-CoA: Hydroxymethylguanosyl coenzyme A reductase
IBDQ-B: Inflammatory bowel disease questionnaire B
IHC: Immunohistochemistry
LR: Local recurrence
mGPS: modified Glasgow prognostic score
MRI: Magnetic resonance imaging
mrTRG: magnetic resonance tumour regression grade
NCI CTCAE: National Cancer Institute Common Terminology Criteria for Adverse Events
NHMRC: National Health and Medical Research Council
NLR: Neutrophil-lymphocyte ratio
OS: Overall survival
pathTRG: pathological tumour regression grade
pCRT: preoperative chemoradiation therapy
PET-CT: Positron-emission tomography- computed tomography
PFS: Progression-free survival
PR: Partial response
PTV: Planning target volume
RT: Radiation therapy
SCRT: Short course radiation therapy
SIM: Simvastatin
Tregs: Regulatory T-cells
US: United States of America
